# The First Selective Screening for Type 1 Diabetes in a Pediatric Population in Bulgaria

**DOI:** 10.3390/jcm15083075

**Published:** 2026-04-17

**Authors:** Natasha Yaneva, Meri Petrova, Adelina Yordanova, Trifon Popov, Margarita Arshinkova, Dobroslav Kyurkchiev, Ekaterina Kurteva

**Affiliations:** 1 Clinic for Pediatric Endocrinology, Diabetes and Metabolic Diseases, University Children’s Hospital “Prof. Ivan Mitev”, 1606 Sofia, Bulgaria; 102268@students.mu-sofia.bg (T.P.); marshinkova@medfac.mu-sofia.bg (M.A.); 2 Department of Pediatrics, Medical Faculty, Medical University of Sofia, 1431 Sofia, Bulgaria; 3 Laboratory of Clinical Immunology, University Hospital St. Ivan Rilski, Department of Clinical Immunology, Medical Faculty, Medical University of Sofia, 1431 Sofia, Bulgaria; meri.petrova1234@gmail.com (M.P.); adelina.d.yordanova@gmail.com (A.Y.); dkyurkchiev@medfac.mu-sofia.bg (D.K.); e.kurteva@medfac.mu-sofia.bg (E.K.)

**Keywords:** type 1 diabetes, islet autoimmunity, screening feasibility, early diagnosis, pediatric diabetes, disease prevention, Bulgaria, pilot study

## Abstract

**Background:** Screening for presymptomatic type 1 diabetes (T1D) reduces the risk of diabetic ketoacidosis (DKA) and allows for early intervention with disease-modifying therapies. Despite the rising incidence of T1D in Bulgaria, screening initiatives remain limited. This pilot study aims to evaluate the feasibility of selective T1D screening in high-risk children and identify potential clinical associations with islet autoimmunity. **Methods:** The study targeted a recruitment of 250 children aged 0–18 years (200 with a relative with T1D and 50 without). Screening for islet autoantibodies (AABs), including glutamic acid decarboxylase (GADA), insulin (IAA), insulinoma-associated-2 (IA-2A), zinc transporter-8 (ZnT8A), and islet cell cytoplasmic autoantibodies (ICAs), was performed via chemiluminescence immunoassay (CLIA). Participants testing positive for one or more AABs were scheduled for longitudinal immunological and metabolic follow-up to evaluate the persistence of autoimmunity and disease progression. **Results:** Between October 2024 and February 2026, the pilot study recruited 210 participants (84% of the 250 target), including 160 children with a relative (target 200) and 50 without a family history of T1D (target 50). Within the high-risk group, seven children (4.4%) tested positive for a single autoantibody (3 GADA, 2 ZnT8A, 1 IA-2A, and 1 IAA), while no autoantibodies were detected in the group without a relative. No cases of multiple autoantibody positivity or stage 3 T1D were identified in either group. Furthermore, no statistically significant associations were observed between autoantibody positivity and secondary factors, including breastfeeding, allergic status, a high-glycemic diet, frequent illness, and personal history of autoimmune disease. **Conclusions:** The findings validate the feasibility of selective T1D screening in Bulgaria, driven by high public interest and successful recruitment across both high-risk and general population cohorts. While this exploratory study found no significant clinical correlations, it establishes a vital roadmap for larger, longitudinal research. Ultimately, this pilot framework provides a scalable model for implementing standardized early detection to reduce the burden of T1D on the national healthcare system.

## 1. Introduction

Type 1 diabetes (T1D) is a chronic autoimmune disease resulting from complex interactions between genetic, environmental, and immune factors [1]. Its progression is characterized by four distinct stages based on the presence of islet autoantibodies (AABs) and glycemic status [2,3]. While the detection of a single AAB indicates a moderate risk, the presence of two or more AABs is highly predictive of clinical disease, with nearly all such individuals progressing to insulin dependence over time [4,5].

Screening programs for islet-specific AABs effectively identify presymptomatic T1D, allowing for early intervention up to 15 years before clinical symptoms appear [4,6,7]. Beyond reducing the rates of diabetic ketoacidosis (DKA) and its associated complications, these programs improve quality of life and provide a window for disease-modifying therapies, such as teplizumab, which can significantly delay the onset of clinical T1D [8,9,10,11,12,13,14,15].

Current screening strategies have evolved from targeting high-risk individuals with a family history to broader general population or genetic-risk-stratified approaches. While family-based screening has a higher per-test yield, it misses up to 90% of future T1D cases, prompting the international momentum toward large-scale general population initiatives [16,17,18,19]. Recent global programs have successfully demonstrated the feasibility of such large-scale screening [20,21,22,23,24].

Despite these global advances, Bulgaria currently lacks an established nationwide screening infrastructure for type 1 diabetes. The epidemiology of T1D in the country follows a rising trend consistent with broader European patterns of increasing autoimmune conditions. Recent data indicate an annual incidence rate of approximately 10–15 cases per 100,000 children in Bulgaria [25]. Considering the local healthcare structure and the urgent need to enhance public awareness, we initiated a pilot screening study. This study focuses primarily on children with first-degree relatives with T1D, who are at significantly higher risk, alongside a general population control group to evaluate the feasibility of larger-scale screening efforts.

The primary objectives of this study are to evaluate the feasibility of T1D screening in Bulgaria, facilitate early diagnosis to reduce the incidence of diabetic ketoacidosis, and establish a framework for the follow-up and education of children positive for islet autoantibodies. This article presents the initial results of this first-of-its-kind selective screening program in the country. Furthermore, we explore potential associations between AAB positivity and secondary environmental or lifestyle factors, including breastfeeding duration, dietary habits, and clinical history.

## 2. Materials and Methods

### 2.1. Study Design and Setting

This is a prospective, longitudinal, single-centre study. While the initial recruitment target was 250 children, the study successfully enrolled 210 participants aged 0–18 years between October 2024 and February 2026. The study cohort includes 160 children with a first-degree relative (a sibling or parent) with T1D (target 200) and 50 control subjects from the general population (target 50) with no family history of the disease. Participants were recruited from across Bulgaria via media announcements (radio, television, and social media), as well as through the practices of pediatric endocrinologists and general practitioners.

### 2.2. Participants and Eligibility Criteria

The inclusion criteria for the study were as follows:

- Age between 0 and 18 years;
- Presence of at least one first-degree relative (parent or sibling) with T1D (for the high-risk cohort);
- Absence of a personal or family history of T1D (for the control group);
- Provision of written informed consent from parents or legal guardians.

The exclusion criteria for both groups were as follows:

- Known history of other types of diabetes (type 2 diabetes, MODY, or secondary diabetes due to pancreatic disease);
- Current use of systemic corticosteroids or other medications known to affect glucose metabolism or immune response;
- Pregnancy at the time of screening;
- Presence of acute infectious diseases or major systemic illnesses at the time of blood sampling;
- Lack of informed consent.

### 2.3. Screening and Laboratory Procedures

Each participant underwent a comprehensive medical history review, physical examination, and laboratory screening for islet AABs, including insulin autoantibodies (IAAs), glutamic acid decarboxylase autoantibodies (GADAs), insulinoma-associated-2 autoantibodies (IA-2As), zinc transporter-8 autoantibodies (ZnT8As), and islet cell cytoplasmic autoantibodies (ICAs). To confirm the persistence of autoimmunity, participants testing positive at the initial screening underwent repeat testing within a 3- to 6-month interval. This follow-up ensured that only persistent antibody positivity, a key predictor of progression to clinical T1D, was considered in the final analysis. Clinical stages of T1D were diagnosed according to the standardized criteria of the American Diabetes Association (ADA) [26].

A chemiluminescent immunoassay (CLIA) was employed to quantitatively detect pancreatic islet autoantibodies associated with T1D, including IAA, GADA, IA-2A, ZnT8A, and ICA. Serum samples were collected and processed according to standard clinical laboratory procedures. Following blood collection, samples were centrifuged to obtain serum, which was aliquoted to prevent repeated freeze–thaw cycles and stored at −80 °C until analysis.

Autoantibodies were measured using a fully automated MAGLUMI X3 CLIA analyzer in accordance with the manufacturer’s instructions. In this assay, recombinant or purified antigens (insulin, GAD65, IA-2, ZnT8, or islet cell antigens), immobilised on paramagnetic microparticles, are mixed with patient serum and bind to the corresponding autoantibodies. After incubation, unbound components are removed by magnetic separation washing. A chemiluminescent substrate (secondary antibody labelled with a chemiluminescent marker) is then added, which reacts with an enzyme linked to the immune complex. The analyzer measures the emitted light in relative light units (RLUs), which are converted to antibody concentrations using a calibration curve generated from standard controls. The presence and concentration of each autoantibody were interpreted using the assay manufacturer’s cutoff values or validated reference ranges, and results were reported in AU/mL, IU/mL, and U/mL, depending on the antibody measured (Table 1).

### 2.4. Follow-Up and Monitoring Protocol

Children who test positive for one or more islet autoantibodies are scheduled for longitudinal follow-up at intervals of 3 to 6 months. This monitoring schedule aligns with the 2024 international consensus guidance for individuals with pre-stage 3 T1D [27].

Participants with a single persistent autoantibody undergo follow-up that includes checking antibody status and metabolic health. This entails measuring fasting and postprandial blood glucose, glycated haemoglobin (HbA1c), and C-peptide levels. When a participant is found to have two or more autoantibodies, the monitoring protocol transitions to a more intensive schedule: children under 3 years receive an oral glucose tolerance test (OGTT) every 3 months, those aged 3–9 years every 6 months, and those 9 years or older are tested annually [27]. Additionally, at intervals, a continuous glucose monitoring (CGM) system is used for 14 days to detect early glycemic variability.

All screening-positive children are monitored over time using standardized growth and weight charts to track their physical development. Additionally, their families receive comprehensive education on adopting a low-glycemic index diet and emphasising regular physical activity. These interventions are intended to optimise metabolic health, improve glycemic stability, and possibly delay progression toward clinical T1D. Effectiveness is evaluated during follow-up visits through structured clinical discussions and qualitative assessments of dietary adherence and lifestyle modifications.

### 2.5. Counselling and Education Interventions

**Figure 1:**
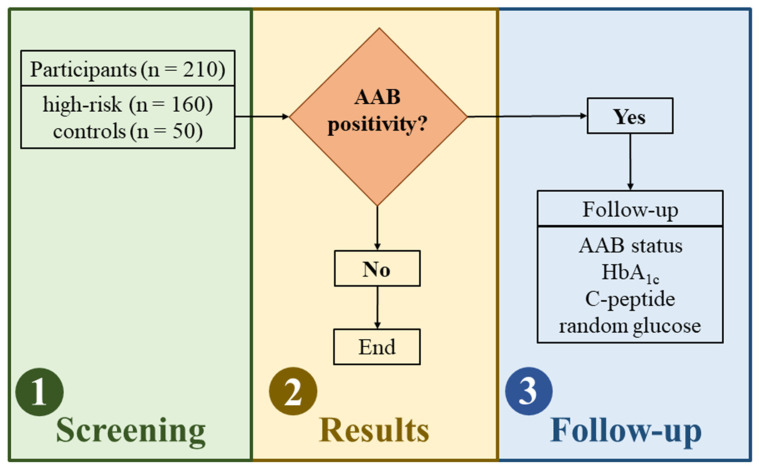
Study design and screening workflow. (Stage 1) A total of 210 participants (160 high-risk children with a first-degree relative with T1D and 50 general population controls) underwent initial screening for islet autoantibodies (AABs) via chemiluminescence immunoassay (CLIA). (Stage 2) Based on the initial results, participants were stratified into AAB-positive and AAB-negative groups. (Stage 3) Children with at least one positive AAB result were scheduled for a 3 to 6-month follow-up to confirm persistent autoimmunity. This longitudinal evaluation included AAB status reassessment and metabolic monitoring, specifically random blood glucose, glycated hemoglobin (HbA1c), and C-peptide levels. No cases of multiple autoantibody positivity were identified in this cohort.

The intervention framework was conducted in three primary phases: (1) initial screening and recruitment, (2) counselling and communication of results, and (3) long-term clinical support. Each stage was designed to provide tailored medical care, continuous monitoring, and structured education for participants and their families. A detailed overview of the study workflow and the sequence of these interventions is presented in Figure 1.

#### 2.5.1. Recruitment and Initial Education

Participants were enrolled through a combination of recruitment strategies, including direct outreach during clinical visits and broader dissemination via national media and social networks. All potential participants received an educational booklet detailing the stages of T1D, the clinical benefits of early diagnosis, and an overview of current disease-modifying therapies. Prior to enrollment, families received comprehensive counselling on study participation, either in person or by telephone, to ensure a clear understanding of the screening workflow.

#### 2.5.2. Results Communication and Lifestyle Counseling

Upon the availability of laboratory findings, a research physician notified the families via telephone. Participants testing positive for one or more islet AABs were invited to the clinical centre for specialized counselling regarding the risk of progression to Stage 3 T1D. These families received personalized psychological support and evidence-based lifestyle recommendations, specifically focusing on a low-glycemic index diet and regular physical activity. Furthermore, participants engaged in person-centred, structured education focused on symptom awareness and healthy coping mechanisms to delay disease progression. Comprehensive guidebooks were provided, and a dedicated contact person was assigned to each family for ongoing clinical inquiries.

#### 2.5.3. Long-Term Support and Education Maintenance

The final phase of the intervention focused on providing longitudinal support for AAB-positive participants. Beyond clinical monitoring, these families received continuous medical guidance and psychological support to maintain adherence to lifestyle modifications. This stage ensured that parents were equipped to recognize early symptoms of hyperglycemia and manage the emotional impact of their child’s increased risk for T1D progression. For families of participants who tested negative, the support focused on ongoing symptom awareness and education regarding the importance of future screening if clinical signs of diabetes mellitus develop.

### 2.6. Statistical Analysis

Statistical analysis was performed using IBM SPSS Statistics (version 26.0; IBM Corp., Armonk, NY, USA). Descriptive statistics were used to summarise the screening data. Categorical variables are expressed as frequencies and percentages. The normality of continuous variables was assessed using the Shapiro–Wilk test, visual inspection of histograms and Q-Q plots, and evaluation of skewness. Continuous variables are presented as mean ± standard deviation (SD) for normally distributed data and as median (interquartile range, IQR) for non-normally distributed data. For comparisons between two independent groups, an independent-samples *t*-test was used for normally distributed variables, while the Mann–Whitney U test was used for non-normally distributed variables. Levene’s test was used to assess variance homogeneity. Effect sizes were estimated using Cohen’s d, calculated as *d* = *M*_1_ − *M*_2_/*SD_pooled_*. For the comparison of categorical values, the chi-square (χ^2^) test or Fisher’s exact test was used, as appropriate. Statistical significance was set at *p* < 0.05.

### 2.7. Ethical Considerations

The study was conducted in accordance with the ethical standards of the Declaration of Helsinki and national research ethics guidelines. The protocol was approved by the Institutional Review Board and the Research Ethics Committee of the authors’ affiliated university, with Protocol № 15 dated 23 July 2024. Written informed consent was obtained from the legal guardians of all participants prior to their inclusion in the study.

Participant privacy and data confidentiality were strictly maintained in accordance with Good Clinical Practice (GCP) standards. No financial compensation was provided to the participants or their families for their involvement in this research.

## 3. Results

The pilot study recruited 210 participants (84% of the 250 target), including 160 children with a family history of T1D (target 200) and 50 without a family history of T1D (target 50). The screening was conducted between October 2024 and February 2026, with 210 children enrolled in the study. The participants were divided into two cohorts: the high-risk group (Group 1; *n* = 160, 76.2%), comprising individuals with a first-degree relative with T1D, and the control group (Group 2; *n* = 50, 23.8%), consisting of children without a family history of T1D.

Table 2 summarizes the baseline characteristics of the study participants. The high-risk cohort (Group 1) comprised 83 girls (51.9%) and 77 boys (48.1%), with a mean age of 8.54 ± 4.72 years (range: 0.8–18.0 years). Although the age distribution in Group 1 showed a slight deviation from normality (Shapiro–Wilk test, *p* = 0.001), visual inspection of histograms and Q-Q plots, along with a skewness value of 0.153, indicated an approximately symmetric distribution, justifying the use of parametric testing.

The control group (Group 2) consisted of 25 females (50.0%) and 25 males (50.0%), with a mean age of 8.20 ± 4.46 years (range: 1.0–17.0 years) and a symmetric distribution (Shapiro–Wilk test, *p* = 0.066; skewness 0.211). No statistically significant differences were observed between the two groups regarding mean age (*t* (208) = 0.45, *p* = 0.652; 95% CI [−1.15, 1.83]; Cohen’s *d* = 0.056) or sex distribution (χ^2^ (1) = 0.054, *p* = 0.817).

Islet AAB positivity was observed in 4.6% (5/109) of breastfed children compared to 3.9% (2/51) of those without a history of breastfeeding (Table 3), showing no significant association (Fisher’s exact test, *p* = 1.000). Although all seven AAB-positive children reported an increased intake of high-glycemic index (GI) foods, no statistically significant link was found between high-GI carbohydrate consumption and AAB status (Fisher’s exact test, *p* = 0.199). Regarding delivery mode, three AAB-positive children were delivered via C-section, but this did not significantly influence AAB positivity (Fisher’s exact test, *p* = 0.466). Furthermore, AAB prevalence was 6.4% among children with allergies and 3.5% in those without, but this difference was not statistically significant (Fisher’s exact test, *p* = 0.420). Finally, no significant correlations were identified between AAB positivity and frequent childhood illnesses (*p* = 0.677) or a personal history of autoimmune diseases (*p* = 0.239).

**Figure 2:**
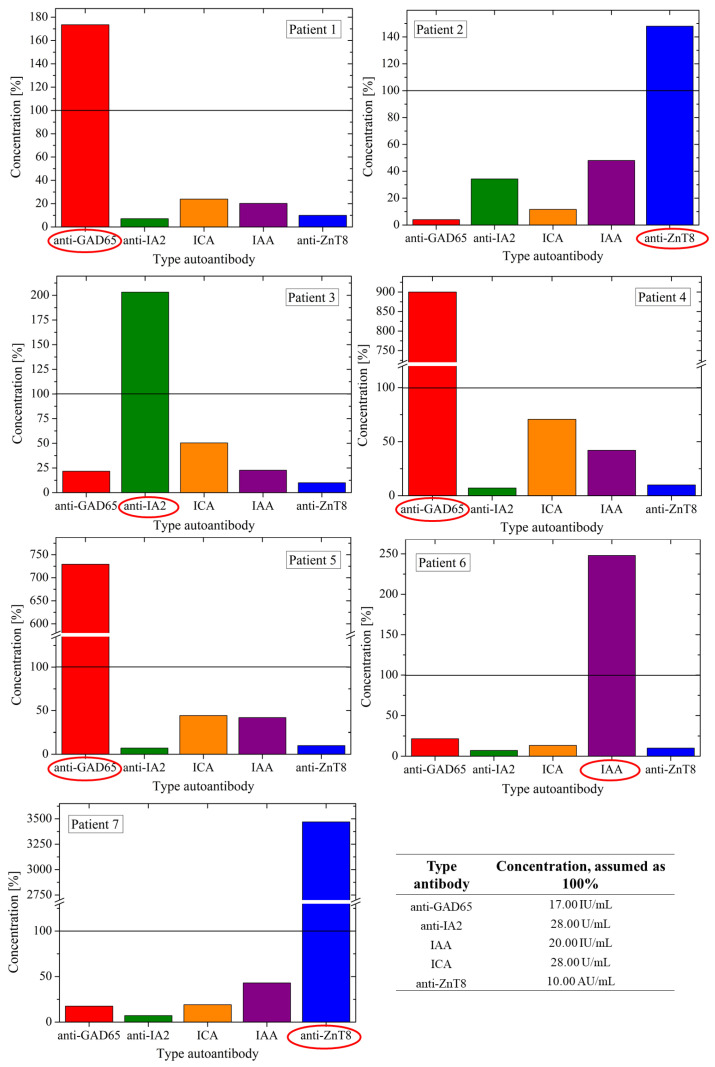
Distribution and relative concentrations of pancreatic islet autoantibodies (AABs) in AAB-positive participants (*n* = 7). Bar charts represent the relative concentrations (%) of five T1D-specific AAB: GADA (red), IA-2A (green), ICA (orange), IAA (purple), and ZnT8A (blue). Concentrations are expressed as percentages relative to the assay-specific reference thresholds (100%), defined as follows: GADA, 17.00 IU/mL; IA-2A, 28.00 U/mL; IAA, 20.00 IU/mL; ICA, 28.00 U/mL; and ZnT8A, 10.00 AU/mL. The horizontal dashed line indicates the 100% clinical cutoff; values above it indicate positive AAB levels. The detected positive AAB for each participant is highlighted with a red ellipse.

The results of the islet AAB screening for all seven positive participants are summarized in Figure 2. The most prevalent AAB identified was anti-GAD65, found in three of the seven children (1.9% of the high-risk cohort). Additionally, two children tested positive for anti-ZnT8 (1.3%), one for anti-IA-2 (0.6%), and one for IAA (0.6%). No AAB positivity was detected among participants in the general population control group.

## 4. Discussion

### 4.1. Feasibility and Recruitment Success

The pilot study confirms the feasibility and high acceptability of a selective T1D screening model in Bulgaria, achieving an overall recruitment rate of 84% (210/250). Notably, the 100% enrollment rate in the general population cohort (50/50) demonstrates significant public interest in early detection, even in the absence of a known family history. However, it is important to acknowledge that approximately 90% of T1D cases occur in children without a family history of the disease. While focusing on first-degree relatives is a practical entry point for pilot initiatives due to their higher relative risk, selective screening alone has a limited reach in reducing the overall national incidence of diabetic ketoacidosis. In contrast, population-wide screening strategies, such as the Fr1da (Germany) or ASK (USA) studies, offer a more comprehensive approach by identifying at-risk children who would otherwise be missed [22,28]. Our findings, particularly the strong engagement from the general population, suggest that Bulgaria is well-positioned to transition from a selective model to a more inclusive, population-wide framework to maximize public health impact.

While the high-risk group reached 80% of its target (160/200), this cohort remains highly representative of the Bulgarian pediatric population. These results validate that the screening infrastructure, from media outreach to clinical sampling, is practical and scalable for future national implementation.

From a health-economic perspective, while population-wide screening is more comprehensive, our selective screening model offers a cost-effective entry point for resource-limited settings. By identifying high-risk individuals and providing early education, this approach can potentially reduce the long-term clinical and financial burden associated with diabetic ketoacidosis and unscheduled hospitalizations.

### 4.2. Study Limitations

Several limitations of this pilot study should be acknowledged. First, the relatively small cohort size (*n* = 210) and the small subset of antibody-positive individuals (*n* = 7) render this study exploratory and hypothesis-generating. Consequently, the lack of significant clinical correlations and the absence of multiple autoantibody-positive cases likely reflect limited statistical power rather than an absence of biological risk or disease progression, as identifying rare immunological events requires larger, multicenter cohorts.

Second, the size of the control group (*n* = 50) reflects the prioritization of resources toward the high-risk cohort to maximize feasibility within this pilot framework. Parents in the high-risk group are often more knowledgeable about T1D and highly motivated to participate, which may introduce a selection bias. While this convenience sampling limits the representativeness of the control group relative to the general Bulgarian population, it allowed for a focused evaluation of the screening infrastructure among those most likely to engage with early detection initiatives.

Third, genetic variability and HLA typing were not performed in this cohort. Given that the interaction between high-risk genotypes and environmental triggers shapes the immune response differently across populations, the absence of immunogenetic markers limits the depth of our risk stratification [29]. Furthermore, the clinical overlap between classic T1D and Latent Autoimmune Diabetes in Adults (LADA) remains a challenge, as GADA and other autoantibodies can fluctuate, requiring careful interpretation to avoid diagnostic confusion [30,31].

Finally, metabolic factors such as insulin resistance and BMI were not a primary focus. While markers such as the Triglycerides/HDL ratio, combined with HbA1c, are effective tools for identifying metabolic syndrome in autoimmune diabetes, the majority of our participants maintained a normal BMI [32]. Despite these limitations, this selective screening represents a necessary first step in the Bulgarian context, providing a preliminary foundation for larger, population-wide screening strategies.

Results from the CLIA testing should be used in conjunction with the patient’s medical history, clinical examination and other findings. If the AAB screening results are inconsistent with clinical evidence, additional testing is needed to confirm the result. Heterophilic antibodies in human serum can react with immunoglobulins, interfering with in vitro immunoassays. Patients routinely exposed to animals or animal serum products may be prone to this interference and anomalous values may be observed.

### 4.3. Comparison with Other Screening Studies

In our cohort, the prevalence of AAB positivity among children with a first-degree relative was 4.4%, which is closely aligned with the 4.8% reported by The TrialNet Natural History Study [33]. However, this rate is notably lower than the frequencies observed by Segovia-Gamboa et al. (16.47%) [34] and Incani et al. (11.9%) [35].

Regarding environmental triggers, our findings did not show consistent associations between islet autoimmunity and the evaluated factors. Similar to the DAISY and MIDIA studies [36,37], we found no significant correlation between breastfeeding and the development of autoantibodies. In contrast, the TEDDY study [38], a large international prospective birth cohort, has indicated that respiratory infections may increase the risk of islet autoimmunity, whereas the impact of gastroenteritis varies by age at infection. Contrary to the findings from the TEDDY study and research by Beyerlein et al., Lin et al., and Rasmussen et al. [39,40,41], our data indicate that the frequency of infections (specifically respiratory and enteroviral) was not associated with AAB positivity. This discrepancy may be attributed to the relatively small size of our pilot cohort, which may lack the statistical power to detect subtle environmental triggers. Additionally, geographical and lifestyle variations in the Bulgarian population, including different patterns of early-life pathogen exposure, could influence the role of infections in the initiation of islet autoimmunity compared to Northern European or North American cohorts.

Furthermore, no significant dependence was observed between AAB status and a history of concomitant allergies (including food, drug, pollen, or dust mite allergies). The relationship between T1D and allergic diseases remains complex and often bidirectional, challenging the traditional “Th1/Th2” paradigm, which suggests these conditions are mutually exclusive. While T1D is traditionally categorized as a Th1-mediated autoimmune disease and allergies as Th2-mediated, emerging evidence suggests that allergy symptoms and IgE sensitization can coexist with, and potentially influence, the development of T1D-related autoantibodies. Indeed, a strong positive association between allergic sensitization (IgE) and T1D has been observed in regions with low incidence rates, supporting the hypothesis of shared underlying pathogenic mechanisms [42].

Finally, we found no statistically significant difference between the seropositive and seronegative groups regarding the presence of associated autoimmune diseases. This contrasts with the findings of Hazime et al. [43] in both pediatric and adult populations in Morocco. However, consistent with the results of Veijola et al. [44], islet autoimmunity in our study was not associated with a family history of other autoimmune conditions.

### 4.4. Psychological and Educational Impact of Screening

Despite the relatively small cohort, the screening program demonstrated significant educational effectiveness. The structured support provided to participating families enhanced their understanding of T1D pathogenesis, risk stratification, and preventive strategies. Although a negative screening result (~96% of cases) does not entirely preclude the future development of T1D, it provided immediate psychological reassurance to the screened families. Furthermore, families with a previously affected member reported increased confidence following the screening; the clear knowledge of their child’s autoantibody status enabled more informed monitoring and created a window for potential early intervention, as outlined in recent consensus guidelines [27].

### 4.5. Longitudinal Follow-Up and Lifestyle Interventions

The study cohort will undergo long-term longitudinal monitoring. Specifically, the seven children with single autoantibody positivity are under ongoing monitoring; following their initial follow-up, they will be re-evaluated at six-month intervals to assess for potential progression to multiple AAB positivity, dysglycemia, or regression to antibody negativity. This monitoring protocol includes reassessment of AAB status and metabolic control, utilizing fasting and postprandial blood glucose, HbA1c and C-peptide levels.

Early lifestyle interventions, emphasizing a low-glycemic index diet and regular physical activity, are recommended as they may support the management and potentially modulate the progression of autoimmune processes. Reducing the metabolic workload on pancreatic beta-cells through dietary modifications may help preserve their functional integrity and potentially mitigate autoimmune triggers. Current evidence suggests that a high intake of sugar and carbohydrates is significantly associated with T1D risk, and increased consumption of sugar-sweetened beverages has been linked to a higher risk of progression from islet autoimmunity to clinical T1D [45].

Furthermore, regular physical activity enhances insulin sensitivity, promotes metabolic stability, and improves overall pancreatic function. These non-invasive lifestyle modifications may potentially be most effective when initiated early in the disease continuum. Timely implementation of these measures requires early AAB detection and expert assessment by a pediatric endocrinologist, underscoring the necessity of standardized, population-wide screening integrated into existing clinical workflows. Given that islet autoantibodies often appear sequentially rather than simultaneously, long-term follow-up is essential to fully delineate the relationship between autoantibody expression and diabetes risk [46]. Finally, incorporating genetic risk scores alongside autoantibody profiles may further refine risk triage and personalized monitoring strategies [47].

## 5. Conclusions

This study demonstrates the feasibility of a selective T1D screening program in Bulgaria, with an 84% recruitment rate and strong public and clinical support. We identified a 4.4% prevalence of single autoantibody positivity among first-degree relatives, providing a key baseline for risk assessment in Bulgarian children. While no statistically significant associations were found between islet autoimmunity and environmental factors such as breastfeeding, allergies, or diet, this may reflect the limited sample size of this pilot study. These results support the development of a scalable infrastructure for early detection.

The success of this initiative highlights the potential of early screening to enable timely metabolic monitoring and proactive lifestyle interventions. Although no cases of multiple autoantibody positivity were found in this phase, ongoing follow-up of antibody-positive participants will assess seroprogression and long-term outcomes. These findings support expanding to larger, multicenter studies to improve risk stratification and promote the integration of standardized T1D screening into Bulgaria’s national healthcare system, with the goal of reducing diabetic ketoacidosis and enhancing long-term clinical outcomes.

## Figures and Tables

**Table 1:** Characteristics of the assays used in this investigation.

Test (Reagent Kit)	MAGLUMI IAA (SNIBE, Shenzhen, China)	MAGLUMI GADA (SNIBE, Shenzhen, China)	MAGLUMI IA-2A (SNIBE, Shenzhen, China)	MAGLUMI ZnT8A (SNIBE, Shenzhen, China)	MAGLUMI ICA (SNIBE, Shenzhen, China)
**Result interpretation**	Negative: <20 AU/mL Positive: ≥20 AU/mL	Negative: <17 IU/mL Positive: ≥17 IU/mL	Negative: <28 U/mL Positive: ≥28 U/mL	Negative: <10 AU/mL Positive: ≥10 AU/mL	Negative: <28 U/mL Positive: ≥28 U/mL
**Analytical measurement range**	8.00–175 AU/mL	5.00–2000 IU/mL	3.50–1000 U/mL	5.00–500 AU/mL	3.00–280 U/mL
**Specimen storage stability**	7 days (2–8 °C) 6 months (−20 °C)	14 days (2–8 °C) 3 months (−20 °C)	14 days (2–8 °C) 6 months (−20 °C)	7 days (2–8 °C) 6 months (−20 °C)	5 days (2–8 °C) 3 months (−20 °C)

**Table 2:** Baseline characteristics of the high-risk cohort (Group 1) and the general population control group (Group 2).

Characteristics	Group 1 (*n* = 160)	Group 2 (*n* = 50)	Total (*n* = 210)
**I. Demographic** **characteristics**			
Sex, female, *n* (%)	83 (51.9%)	25 (50.0%)	108 (51.4%)
Age (years), mean ± SD	8.54 ± 4.72	8.20 ± 4.46	8.46 ± 4.65
BMI Z-score, mean ± SD	0.37 ± 1.15	0.53 ± 1.73	0.41 ± 1.31
**II. Perinatal and** **Early Life History**			
Cesarean delivery, *n* (%)	91 (56.9%)	32 (64.0%)	123 (58.6%)
Non-breastfed, *n* (%)	51 (31.9%)	23 (46.0%)	74 (35.2%)
**III. Clinical history**			
History of allergies, *n* (%)	47 (29.4%)	15 (30.0%)	62 (29.5%)
Infrequent illness history, *n* (%)	118 (73.7%)	26 (52.0%)	144 (68.6%)
Personal history of autoimmune disease, *n* (%)	6 (3.8%)	4 (8.0%)	10 (4.8%)
**IV. Lifestyle and** **Dietary Habits**			
High-glycemic index food intake, *n* (%)	122 (76.2%)	36 (72.0%)	158 (75.2%)
**V. Family History of** **T1D (Group 1 only)**			
Mother, *n* (%)	57 (35.6%)	–	57 (27.1%)
Father, *n* (%)	44 (27.5%)	–	44 (21.0%)
Sibling, *n* (%)	56 (35.0%)	–	56 (26.7%)
Multiple relatives (>1), *n* (%)	3 (1.9%)	–	3 (1.4%)

**Table 3:** Comparison of perinatal and lifestyle factors between AAB-positive and AAB-negative children.

Characteristic	AAB-Negative (*n* = 203)		AAB-Positive (*n* = 7)		*p* Value
	*n*	%	*n*	%	
Cesarean delivery	88	96.7	3	3.3	0.466
Non-breastfed	49	96.1	2	3.9	1.000
High-glycemic index food intake	115	94.3	7	5.7	0.199
History of allergies	44	93.6	3	6.4	0.420
Infrequent illness History	112	94.9	6	5.1	0.677
Personal history of autoimmune disease	5	83.3	1	16.7	0.239

## Data Availability

The original contributions presented in the study are included in the article; further inquiries can be directed to the corresponding author.

## Abbreviations

The following abbreviations are used in this manuscript:

T1D	Type 1 diabetes
AAB	Autoantibody
DKA	Diabetic ketoacidosis
GADA/anti-GAD65	Anti-glutamic acid decarboxylase 65
IA-2A/anti-IA-2	Anti-insulinoma-associated antigen 2
ZnT8A/anti-ZnT8	Anti-zinc transporter 8
ICA	Islet cell autoantibodies
IAA	Insulin autoantibodies
CLIA	Chemiluminescent immunoassay
OGTT	Oral glucose tolerance test
HbA1c	Glycated hemoglobin
